# Self-Reported Side Effects Associated With Selective Androgen Receptor Modulators: Social Media Data Analysis

**DOI:** 10.2196/65031

**Published:** 2025-02-18

**Authors:** Aditya Joshi, Diego Federico Kaune, Phillip Leff, Emily Fraser, Sarah Lee, Morgan Harrison, Moustafa Hazin

**Affiliations:** 1 Department of Internal Medicine Phoenix Health Sciences Campus Creighton University School of Medicine Phoenix, AZ United States; 2 Johns Hopkins University Baltimore, MD United States

**Keywords:** selective androgen receptor modulator, SARM, liver toxicity, social media, data analysis, anabolic, muscle, bone, toxicities, self-report, side effect, retrospective analysis, public post, Reddit, androgen receptor ligands, drug

## Abstract

This study focuses on the prevalence and dangers of selective androgen receptor modulator use through a social media (Reddit) analysis. Physicians should be aware that patients are increasingly seeking medical advice through social media, and adverse events are likely underreported in the literature.

## Introduction

Selective androgen receptor modulators (SARMs) are synthetic androgen receptor ligands designed to have anabolic effects in muscle and bone while minimizing unwanted androgenic effects in areas like the prostate and cardiovascular system [1]. This tissue selectivity makes SARMs an attractive option for treating conditions such as osteoporosis and muscle wasting while avoiding the systemic side effects associated with traditional androgen therapies. However, no SARM has received US Food and Drug Administration approval to date. Studies show SARMs are associated with acute hepatotoxicity [2], and recent case reports indicate long-term testosterone suppression along with nephrotoxicity and cardiotoxicity [3,4]. Abuse of SARMs has become increasingly prevalent due to targeting of both athletes and youth through social media platforms. While reports of SARM abuse and toxicity have been rising, no study has examined adverse events (AEs) and laboratory data using self-reported social media posts.

## Methods

### Overview

**Figure 1:**
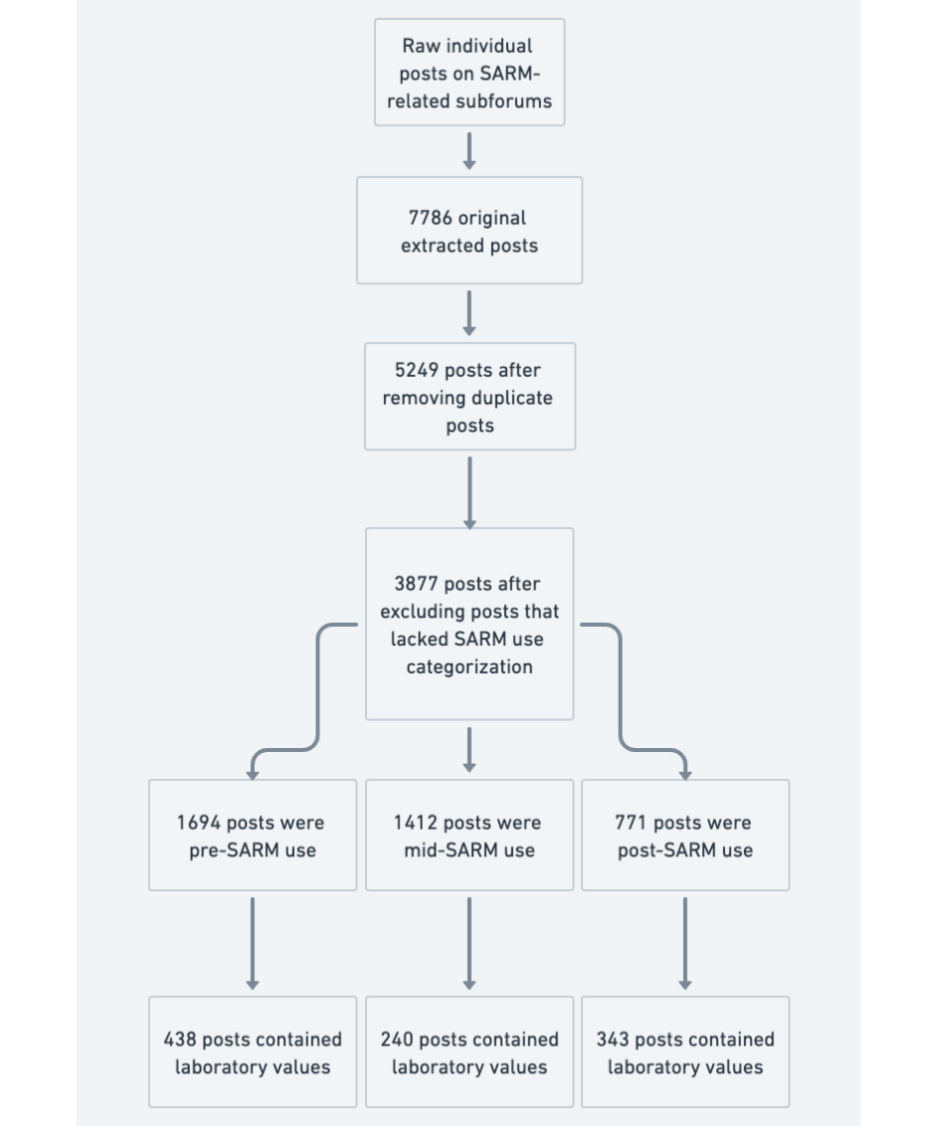
Flowchart of data extraction methodology. Data extraction of posts from selective androgen receptor modulator (SARM)–related subreddits yielded 7786 initial posts. Manual data cleaning removed 2537 duplicates and 1372 uncategorizable posts. The remaining 3877 posts were classified into pre-SARM use (n=1694), mid-SARM use (n=1412), and post-SARM use (n=771). Clinical laboratory values were voluntarily reported in 438 pre-use, 240 mid-use, and 343 post-use posts, enabling analysis of physiological changes associated with recreational SARM use. This retrospective analysis captured real-world SARM use patterns and their associated clinical outcomes in a young male population.

This study involved a retrospective analysis of public posts from March 2015 to November 2023 on Reddit. Data collection used a Python-based script to extract and filter posts from SARM-related subreddits. Posts were identified through a keyword-matching process, using “liver,” “AST,” “ALT,” “lab-work,” “bloodwork,” and “symptoms.” Initial keywords were selected and refined to capture posts reporting laboratory results and hepatic outcomes associated with SARM use. Posts were manually reviewed and systematically excluded if unrelated to SARM use. Posts were categorized based on users’ self-reported stages of SARM use: “precycle,” “midcycle,” and “postcycle” (Figure 1). Demographic data, specific SARMs taken, mentions of concurrent medications, self-reported symptoms, and self-reported laboratory values were extracted. Means of self-reported laboratory values were calculated and compared across stages of SARM use. Female posters were excluded from the calculations of luteinizing hormone, follicle-stimulating hormone, total testosterone, free testosterone, estrogen, and sex hormone–binding globulin.

### Ethical Considerations

To protect user privacy, identifying information was anonymized during data extraction. Data were aggregated to prevent linkage to individual users. No identifiable information was retained or reported. This study was exempt from institutional review board approval and strictly adhered to ethical guidelines for digital data use.

## Results

Initially, 5249 unique posts were extracted, of which 1372 were excluded due to irrelevance or the inability to categorize the stage of SARM use. Of the remaining 3877 posts, 1694 were pre-SARM use, 1412 were mid-SARM use, and 771 were post-SARM use. RAD140 was the most mentioned SARM, with 1389 posts. The mean reported age was 27 (range 13-63, SD 7.9) years. In 418 posts reporting gender, 20 (4.8%) posters identified as female. Significant changes in aspartate aminotransferase, alanine aminotransferase, creatinine kinase, high-density lipoprotein, low-density lipoprotein, total testosterone, and sex hormone–binding globulin were observed before, during, and after SARM use (Table 1 and Multimedia Appendix 1). Of 2183 SARM users, 382 (17.5%) reported using tamoxifen or enclomiphene, and 170 (7.8%) reported using N-acetylcysteine or other hepatoprotective supplements (milk thistle or tauroursodeoxycholic acid) during or after SARM use.

**Table 1:** A comparison of the mean self-reported clinical markers before, during, and after selective androgen receptor modulator (SARM) use. This retrospective analysis examined changes in liver function, kidney function, hormones, and lipid profiles in recreational SARM users who voluntarily reported laboratory results. Of note, the study population consisted primarily of young men. Statistical significance was assessed using paired t tests comparing baseline values to both during-use and post-use periods. *P*<.05 indicates statistical significance. The large SDs in many measurements indicate high variability in individual physiological responses. Normal values refer specifically to men. Women were removed from the analysis of luteinizing hormone, follicle-stimulating hormone, total testosterone, free testosterone, estrogen, and sex hormone–binding globulin.

Clinical markers	Normal values	Before SARM use		During SARM use		*P* value	After SARM use		*P* value
		Posts, n	Self-reported values, mean (SD)	Posts, n	Self-reported values, mean (SD)		Posts, n	Self-reported values, mean (SD)	
Aspartate aminotransferase (U/L)	10-30	121	27.7 (17.6)	89	74.3 (85.5)	<.001	125	83.5 (322.1)	.03
Alanine aminotransferase (U/L)	10-40	143	29.5 (19.8)	99	125.6 (171.9)	<.001	139	108.7 (318.9)	.002
Alkaline phosphatase (U/L)	30-120	84	76.4 (25.2)	43	68.8 (28.7)	.06	78	70.9 (35.5)	.12
Bilirubin (mg/dL)	0.3-1.2	88	1.5 (3.8)	41	1.0 (1.9)	.18	79	1.3 (4.8)	.35
Creatinine (mg/dL)	0.6-1.2	77	1.1 (0.2)	41	1.1 (0.2)	.41	71	1.1 (0.2)	.21
Creatine kinase (U/L)	40-150	9	259.1 (344.6)	11	727.1 (675.2)	.03	8	683.1 (450.7)	.02
Luteinizing hormone (U/L)	1.24-7.8	195	6.1 (17.0)	78	4.8 (3.2)	.25	145	6.0 (7.8)	.48
Follicle-stimulating hormone (U/L)	1.4-15.4	187	4.8 (11.1)	74	3.7 (2.5)	.21	135	4.2 (3.4)	.27
High-density lipoprotein (mg/dL)	≥60	116	44.5 (17.9)	59	31.1 (14.9)	<.001	107	31.3 (14.6)	<.001
Low-density lipoprotein (mg/dL)	<100	115	80.6 (43.4)	54	100.3 (50.5)	.004	108	98.4 (50.7)	.002
Cholesterol (mg/dL)	<200	105	132.0 (49.2)	45	142.8 (61.7)	.13	84	136.4 (56.2)	.28
Triglycerides (mg/dL)	<150	84	50.6 (28.0)	40	67.7 (54.7)	.01	71	57.6 (38.6)	.10
Total testosterone (ng/dL)	270-1070	388	585.5 (233.8)	177	358.6 (325.8)	<.001	291	457.7 (346.3)	<.001
Free testosterone (pg/mL)	66-309	188	90.1 (80.8)	95	94.9 (118.8)	.33	123	86.0 (74.6)	.32
Estrogen (pg/mL)	10-50	171	32.2 (23.4)	85	41.5 (61.7)	.04	136	33.1 (18.9)	.36
Sex hormone–binding globulin (nmol/L)	14-78	146	43.9 (98.8)	82	12.8 (10.7)	.003	108	22.0 (16.7)	.01
Hemoglobin (g/dL)	14-18	58	19.9 (25.4)	24	20.8 (28.5)	.45	51	14.5 (2.9)	.07

## Discussion

Recent years have seen an alarming increase in targeted marketing of SARMs on social media, where they are portrayed as safe and effective [5]. Despite being illegal to sell for human consumption, SARMs are available for purchase through numerous online platforms, including commercial websites and chemical suppliers [6]. This analysis revealed that prior, current, and prospective SARM users frequently discuss their experiences, sources, dosing regimens, perceived benefits, laboratory results, and AEs on social media. Distributors also frequent these platforms to offer advice while marketing their products.

Our analysis revealed serious AEs of SARMs, with hepatotoxicity as a prominent concern. Comparing mean aspartate aminotransferase and alanine aminotransferase values across different stages of SARM use showed significant increases, indicative of drug-induced liver injury. Users attempted to self-manage hepatic complications through N-acetylcysteine, milk thistle, and tauroursodeoxycholic acid supplementation and hormonal imbalances with tamoxifen and enclomiphene. Our findings corroborate previous literature that has reported consistent patterns of hepatic changes attributable to SARM use [7].

This analysis has important limitations. The demographics of the sample predominantly comprised young men, limiting generalizability across gender and age. While self-reported laboratory values offer valuable preliminary insights, interindividual variability and small sample sizes in the reported data present an inherent challenge. To increase the robustness of the findings, future work should integrate traditional clinical data and include sensitivity analysis or weighted modeling. Moreover, since efforts were made to capture a wide net of hepatotoxic-specific data, the extraction methodology may underrepresent other AEs. Nonetheless, our findings highlight the need for increased awareness and regulation to address a growing public health crisis posed by SARM abuse. Until then, physicians should recognize that patients are increasingly seeking medical advice through social media and that AEs are likely underreported in the literature.

## Appendix

Multimedia Appendix 1 Changes in liver function tests with selective androgen receptor modulator (SARM) use. Changes in mean aspartate aminotransferase (AST), alanine aminotransferase (ALT), and alkaline phosphatase levels across 3 time periods: before (black bars), during (light gray bars), and after (dark gray bars) SARM use. Data were collected from self-reported laboratory values posted by young men on online forums. Sample sizes varied for each marker: AST (before: n=121; during: n=89; after: n=125), ALT (before: n=143; during: n=99; after: n=139), and alkaline phosphatase (before: n=84; during: n=43; after: n=78). Statistical significance is indicated by asterisks (**P*<.05, ***P*<.01, ****P*<.001). Values are presented as means with error bars representing SDs. Notable elevations were observed in both AST and ALT during and after SARM use, while alkaline phosphatase remained relatively stable.

## Abbreviations

AE: adverse event
SARM: selective androgen receptor modulator

## Data Availability

The data sets generated during and/or analyzed during this study are available from the corresponding author on reasonable request.
