# Supplementary figures and images for: Self-Reported Side Effects Associated With Selective Androgen Receptor Modulators: Social Media Data Analysis
Source: J Med Internet Res. 2025 Feb 18;27:e65031. doi: 10.2196/65031 (PMC11888096; doi:10.2196/65031)

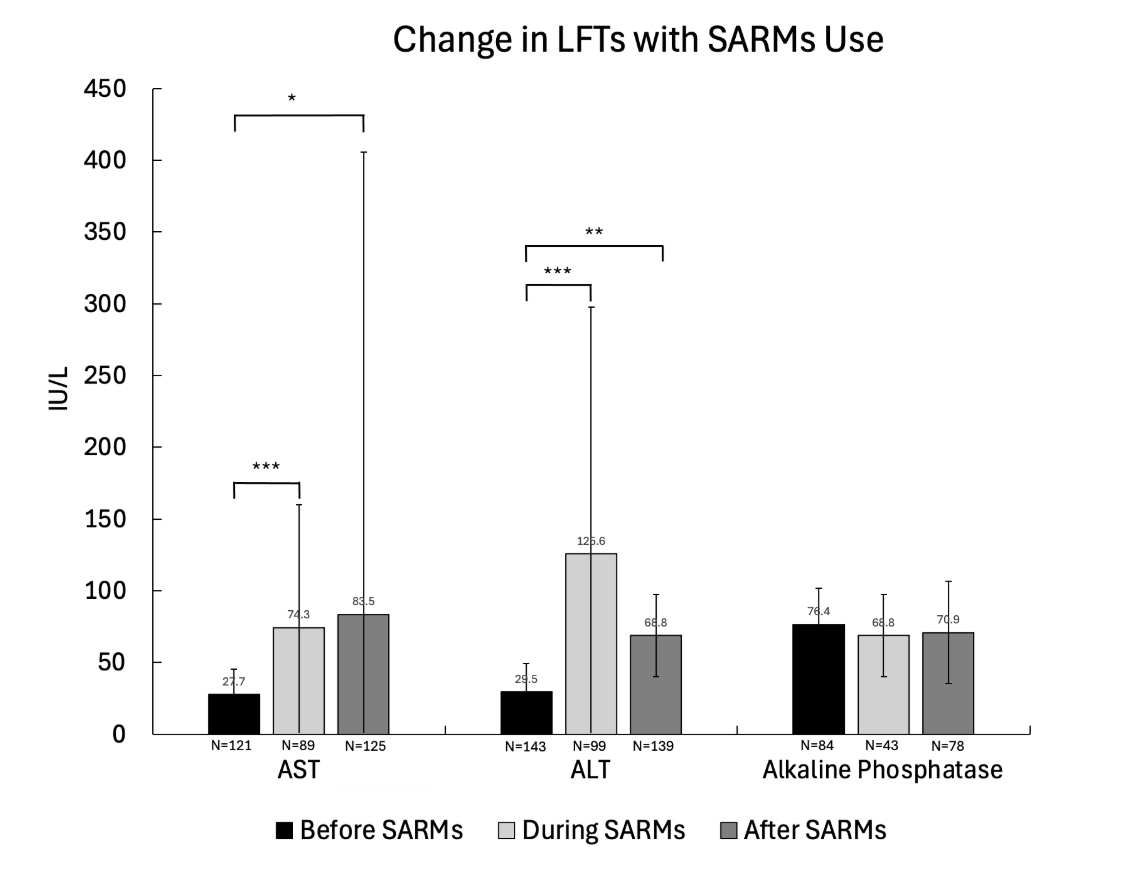

Supplement: Multimedia Appendix 1 [file jmir_v27i1e65031_app1.png]
